# Phase Transition and Electronic Structures of All-*d*-Metal Heusler-Type X_2_MnTi Compounds (X = Pd, Pt, Ag, Au, Cu, and Ni)

**DOI:** 10.3389/fchem.2020.546947

**Published:** 2020-12-11

**Authors:** Mengxin Wu, Feng Zhou, Rabah Khenata, Minquan Kuang, Xiaotian Wang

**Affiliations:** ^1^School of Physical Science and Technology, Southwest University, Chongqing, China; ^2^Laboratoire de Physique Quantique de la Matière et de la Modélisation Mathématique (LPQ3M), Université de Mascara, Mascara, Algeria

**Keywords:** spintronic, electronic structure, DFT, electronic properties, Heusler alloys

## Abstract

In this work, we investigated the phase transition and electronic structures of some newly designed all-*d*-metal Heusler compounds, X_2_MnTi (X = Pd, Pt, Ag, Au, Cu, and Ni), by means of the first principles. The competition between the XA and L2_1_ structures of these materials was studied, and we found that X_2_MnTi favors to feature the L2_1_-type structure, which is consistent with the well-known site-preference rule (SPR). Under the L2_1_ structure, we have studied the most stable magnetic state of these materials, and we found that the ferromagnetic state is the most stable due to its lower energy. Through tetragonal deformation, we found that the L2_1_ structure is no longer the most stable structure, and a more stable tetragonal L1_0_ structure appeared. That is, under the tetragonal strain, the material enjoys a tetragonal phase transformation (i.e., from cubic L2_1_ to tetragonal L1_0_ structure). This mechanism of L2_1_-L1_0_ structure transition is discussed in detail based on the calculated density of states. Moreover, we found that the energy difference between the most stable phases of L1_0_ and L2_1_, defined as Δ*E*_M_ (Δ*E*_M_ = *E*_Cubic_-*E*_Tetragonal_), can be adjusted by the uniform strain. Finally, the phonon spectra of all tetragonal X_2_MnTi (X = Pd, Pt, Ag, Au, Cu, and Ni) phases are exhibited, which provides a powerful evidence for the stability of the tetragonal L1_0_ state. We hope that our research can provide a theoretical guidance for future experimental investigations.

## Introduction

Magnetic shape memory compounds (MSMAs) (O'Handley, 1998) are a new type of intelligent materials which integrates magnetic controlled shape memory and magnetic field-induced strain simultaneously. It can be used as key components of sensors and brakes in the future. MSMAs have both thermoplastic martensitic transformation (Oikawa et al., 2001) and magnetic transformation (Oikawa et al., 2002), and their shape memory effect can be controlled by the magnetic field. That is to say, under the effect of magnetic field, their size or volume will be changed, resulting in a great strain, i.e., the magnetostrictive effect (Populoh et al., 2012). In addition, MSMAs enjoy magnetoresistance (Ullakko et al., 1996) and magnetocaloric (GschneidnerJr et al., 2005) effects, so they have been regarded as a research hot spot in recent years.

Heusler (Graf et al., 2011; Birkel et al., 2013; Ahmadian and Salary, 2014; Kirievsky et al., 2014; Xue et al., 2016; Miranda and Gruhn, 2017; Ghunaim et al., 2018; Li et al., 2019, 2020) compounds belong to intermetallic compounds. Heusler compounds naturally have many excellent properties, such as high Curie temperature (*T*_C_) (Wurmehl et al., 2005), adjustable electronic structure, suitable semiconductor lattice constants, and various magnetic properties. Therefore, Heusler compounds can be seen as good candidates for spin gapless semiconductors (SGSs) (Gao and Yao, 2013; Skaftouros et al., 2013; Wang et al., 2016; Wang X. et al., 2017), thermoelectric materials (Downie et al., 2013; Huang et al., 2018; Mallick and Vitta, 2018), shape memory compounds (SMAs) (Aksoy et al., 2009; Li et al., 2018), half metals (Shigeta et al., 2018; Singh and Gupta, 2019; Hao et al., 2020), and topological insulators (Hou et al., 2015; Lin et al., 2015). Because Heusler compounds possess excellent properties, they have been regarded as a research hot spot over the past 100 years. To date, researchers have discovered thousands of Heusler compounds. Heusler compounds roughly can be divided into three structures, namely, full Heusler (Wang X. T. et al., 2017), half Heusler (Silpawilawan et al., 2017), and quaternary Heusler (Cui et al., 2019), whose stoichiometric compositions are X_2_YZ, XYZ, and XYMZ, respectively. X, Y, and M are usually transition elements, while the Z atom is a main group element. In recent years, a new type of Heusler compounds has been found by researchers, and this type of Heusler compounds is named as all-*d*-metal Heusler compounds (Wei et al., 2015).

In the early 1990s, all-*d*-metal Heusler compounds Zn_2_AuAg and Zn_2_CuAu (Muldawer, 1966) were synthesized experimentally, which proved that all-*d*-metal Heusler compounds can be successfully prepared. This interesting study opened up a new direction for the research of Heusler compounds. All-*d*-metal Heusler compounds are different from common Heusler compounds in that they are composed of transition metal elements without the participation of main group elements. Compared with common Heusler compounds, all-*d*-metal Heusler compounds have the following advantages: (1) all-*d*-metal Heusler compounds do have not many restrictions on atomic site preference, so they can show more phase space and versatility than traditional materials; (2) they have high strength and toughness; and (3) in addition to magnetic phase transitions, all-*d*-metal Heusler compounds also have many untouched physical properties, such as spintronics properties.

Recently, an effective method, i.e., adjusting composition, was proposed by Tan et al. (2019) to make regular the phase transition of all-*d*-metal Heusler compounds. In their work, the atomic ordering, structural stability, tetragonal deformation, magnetism, and electronic structures of the Mn–Ni–V system, including Mn_2−x_Ni_1.5+x_V_0.5_, Mn_2−x_Ni_1+x_V, and Mn_2−x_Ni_0.5+x_V_1.5_ compounds, are studied by employing the first principles. They stated that the tetragonal phase is more stable than the cubic phase for these all-*d*-metal Heusler compounds: MnNi_2_V, Mn_1.25_Ni_1.75_V, MnNi_2.5_V_0.5_, and Mn_0.5_Ni_2_V_1.5_.

Based on the above information, we focus on the electronic structures and phase transition of all-*d*-metal Heusler compounds X_2_MnTi (X = Pd, Pt, Ag, Au, Cu, and Ni) with the help of the first principles. To the best of knowledge, the physical properties of the X_2_MnTi system have not been studied yet by other researchers up to now. We will reflect that a L2_1_-L1_0_ phase transition can be found in all-*d*-metal Heusler compounds X_2_MnTi (X = Pd, Pt, Ag, Au, Cu, and Ni) under the tetragonal distortion. The effect of uniform strain on the cubic–tetragonal transition for this X_2_MnTi system was also discussed in detail in this work. The mechanism of the L2_1_-L1_0_ phase transition is discussed according to the calculated density of states in both cubic and tetragonal phases, and finally, we will further prove the stability of the tetragonal phase by calculating the phonon spectrum of the tetragonal L1_0_ phase.

## Computational Methods

Electronic structure calculations were performed using density functional theory (DFT), within the VASP code (Hafner, 2007). The exchange–correlation potential is treated by using the generalized gradient approximation (GGA) (Perdew et al., 1996) with the Perdew–Burke–Ernzerhof (PBE) (Perdew et al., 1998) functional. We also use the projection enhanced wave (PAW) (Kresse and Furthmüller, 1996) method to deal with the interaction between the ion nucleus and valence electron. In the calculations, the cutoff energy was set at 450 eV. A Monkhorst-Pack special 12 × 12 × 12 k-point mesh was used in the Brillouin zone (BZ) integration. The unit cell was optimized until the force and total energy were <0.001 eV/Å and 1 × 10^−5^ eV, respectively. The crystal models of cubic L2_1_ phase and tetragonal L1_0_ phase of Heusler alloys are built *via* VESTA. To calculate the dynamical stabilities of these alloys, phonon dispersion is obtained by means of the force-constants method using Nanodcal code. For the L2_1_-type X_2_MnTi alloys, two magnetic states are considered: one is the ferromagnetic state with spin orderings of X-1, X-2, Mn, and Ti that are ↑*↑↑↑*; the other one is the antiferromagnetic state with spin orderings of X-1, X-2, Mn, and Ti that are ↓*↓↑↓*, respectively.

## Results and Discussion

### The Crystal and Magnetic Structures of the X_2_MnTi (X = Pd, Pt, Ag, Au, Cu, and Ni) Heusler Compounds

**Figure 1:**
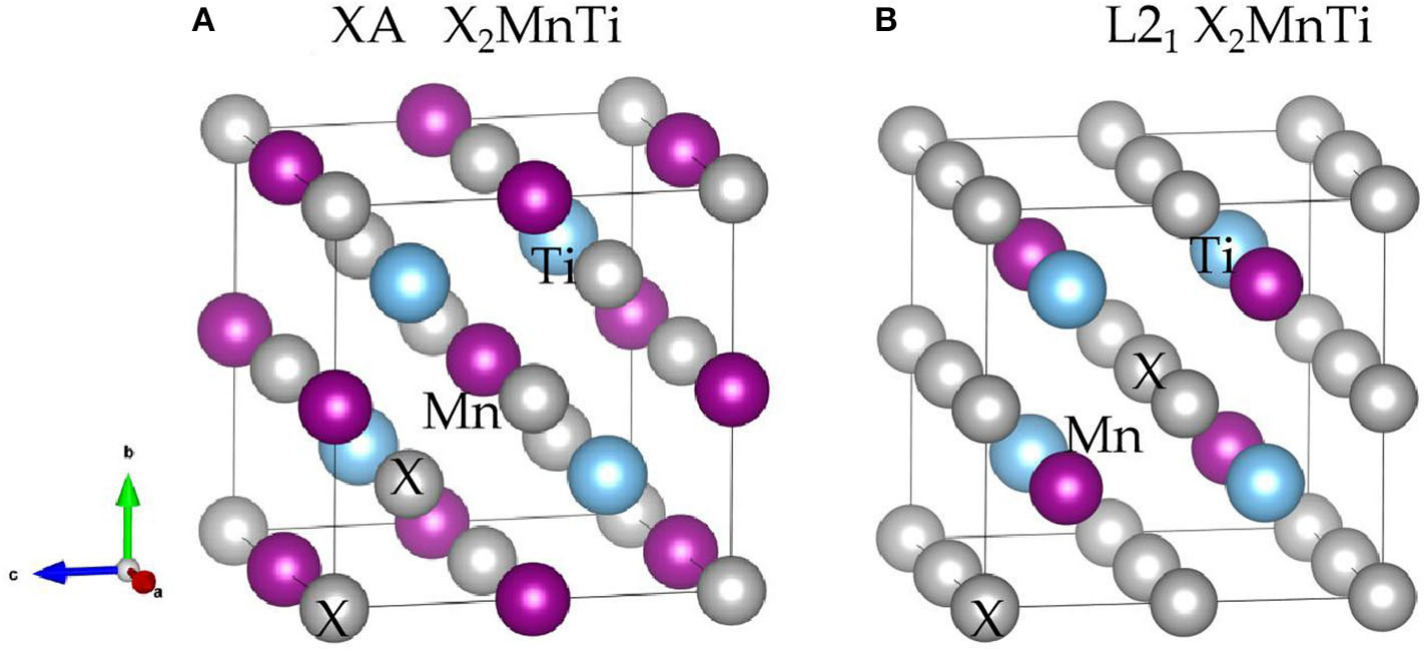
Crystal structure of the **(A)** XA-type X_2_MnTi and **(B)** L2_1_-type X_2_MnTi (X = Pd, Pt, Ag, Au, Cu, and Ni); X is highlighted by gray balls, Ti is marked by blue balls, and Mn is marked by purple balls, respectively.

Heusler compounds, X_2_YZ, enjoy a highly ordered cubic structure (Han et al., 2019a). There are generally four different positions in a primitive cell, namely, A (0, 0, 0), B (0.25, 0.25, 0.25), C (0.5, 0.5, 0.5), and D (0.75, 0.75, 0.75), respectively. The transition metal elements X and Y occupy the A, B, and C positions, and the main group element Z is preferred to occupy the D position. Different occupation positions of X and Y atoms will lead to different structures, namely, L2_1_ and XA structures (Suzuki and Kyono, 2004) (as shown in Figures 1A,B). In the former, two X atoms occupy A and C positions, and Y and Z atoms enter B and D positions, respectively; in the latter, two X atoms occupy A and B positions, and Y and Z atoms are located at C and D positions, respectively.

**Figure 2:**
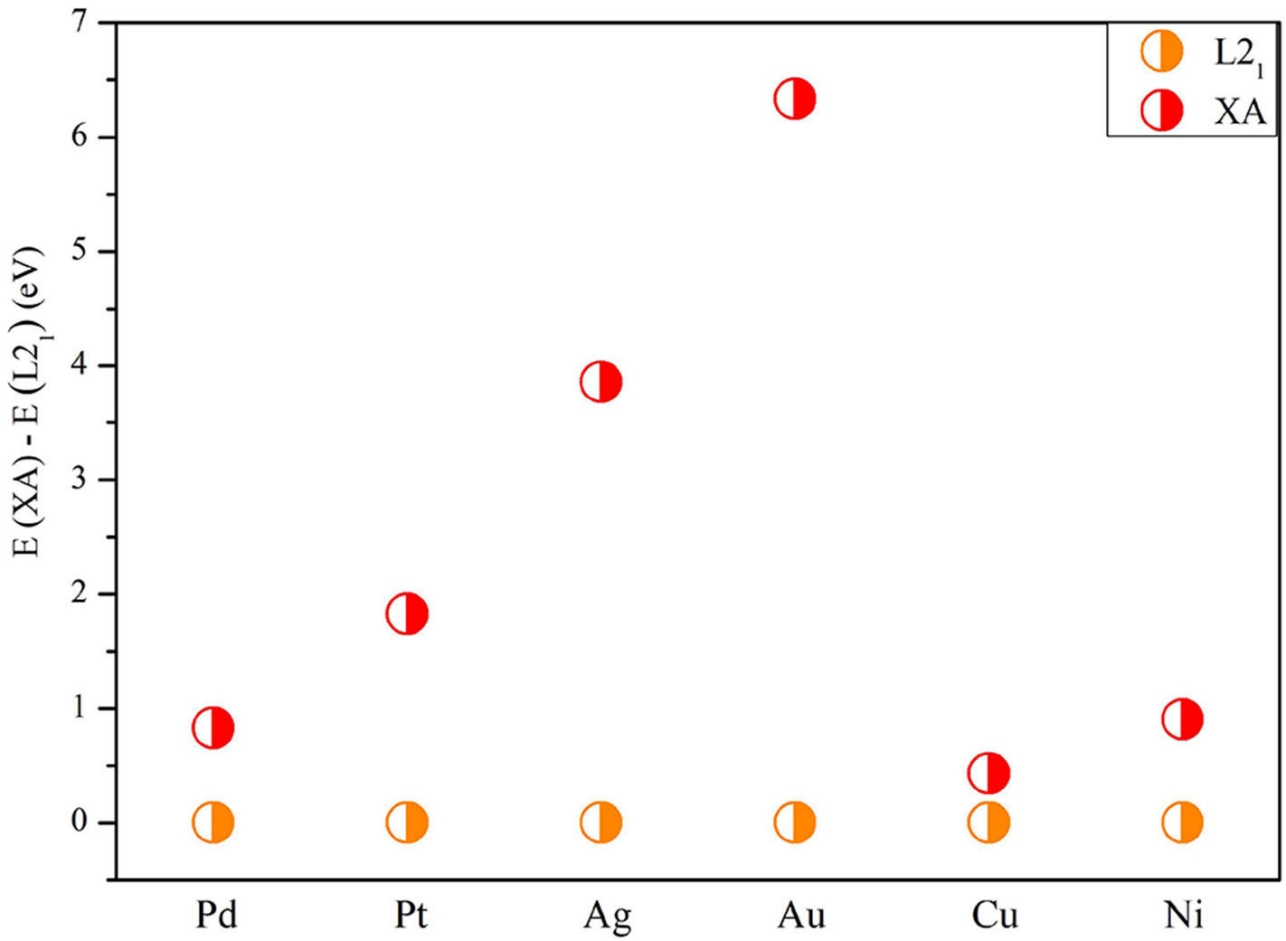
Total energy difference *E*(XA) – *E*(L2_1_) of X_2_MnTi under the L2_1_ and XA structure (in FM state) with the energy of L2_1_ set as 0 eV for reference.

**Figure 3:**
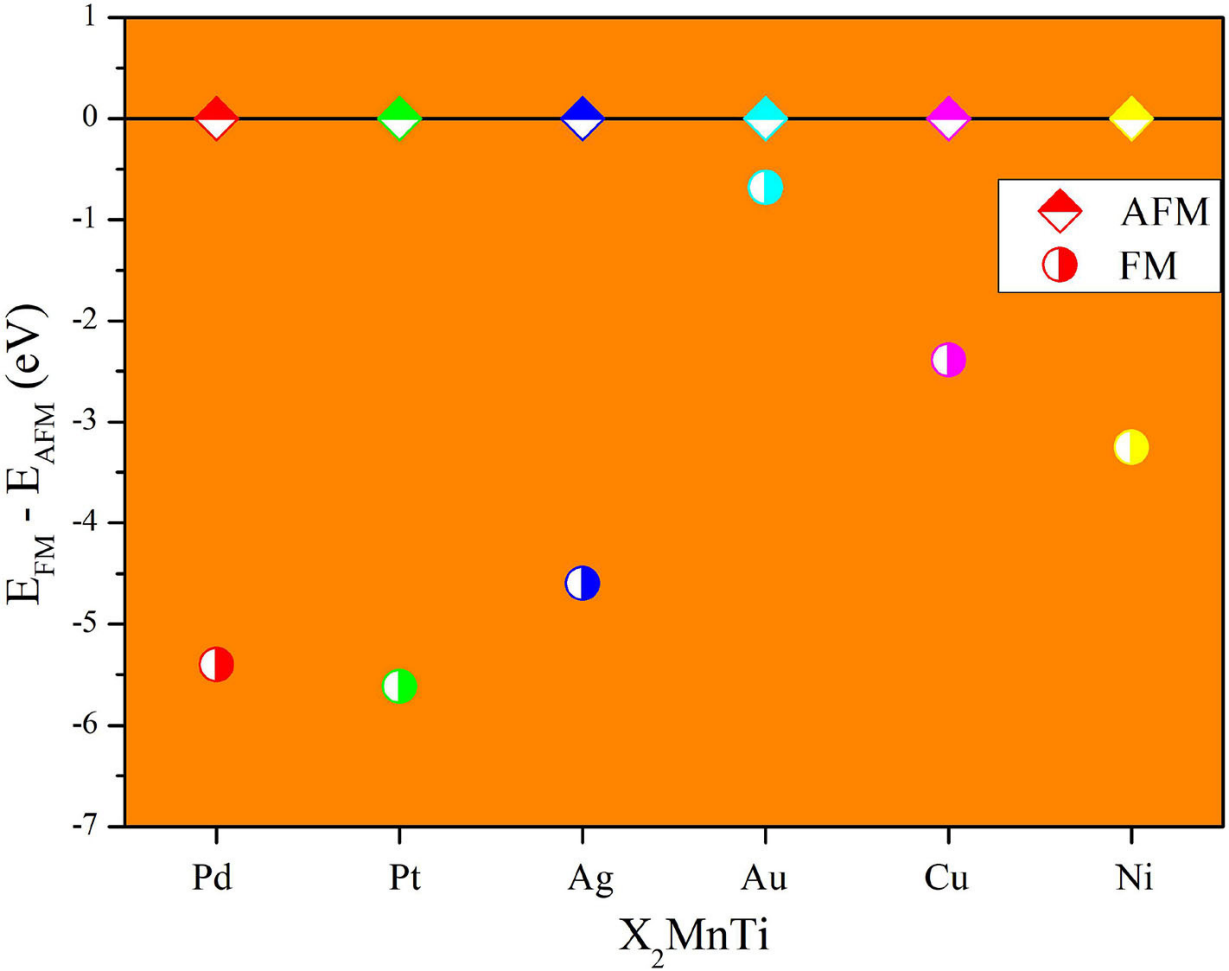
Total energy difference (*E*_FM_ – *E*_AFM_) of different magnetic states (AFM and FM) of X_2_MnTi (L2_1_ structure) with the total energy of AFM set as 0 eV.

Next, we studied the competition between the XA structure and the L2_1_ structure of X_2_MnTi. In Figure 2, the total energy of X_2_MnTi at the ground state with different structures (L2_1_ and XA) is determined, and we set the ground state energy of the L2_1_ structure as 0 eV as reference. One can clearly see that, for X_2_MnTi, the total energy of XA is higher than L2_1_, reflecting that the most stable ordered structure is L2_1_ for the X_2_MnTi system. In Figure 3, we further give a comparison of the total energy of two magnetic states [ferromagnetic (FM) and antiferromagnetic (AFM)] for the L2_1_-type X_2_MnTi. We set the total energy of AFM to 0 eV as reference. As shown in Figure 3, the energy of X_2_MnTi in the FM structure is lower than 0, which indicates that the X_2_MnTi tends to exhibit the FM magnetic state.

### The L2_1_-L1_0_ Phase Transition of All-*d*-Metal Heusler-Type X_2_MnTi

**Figure 4:**
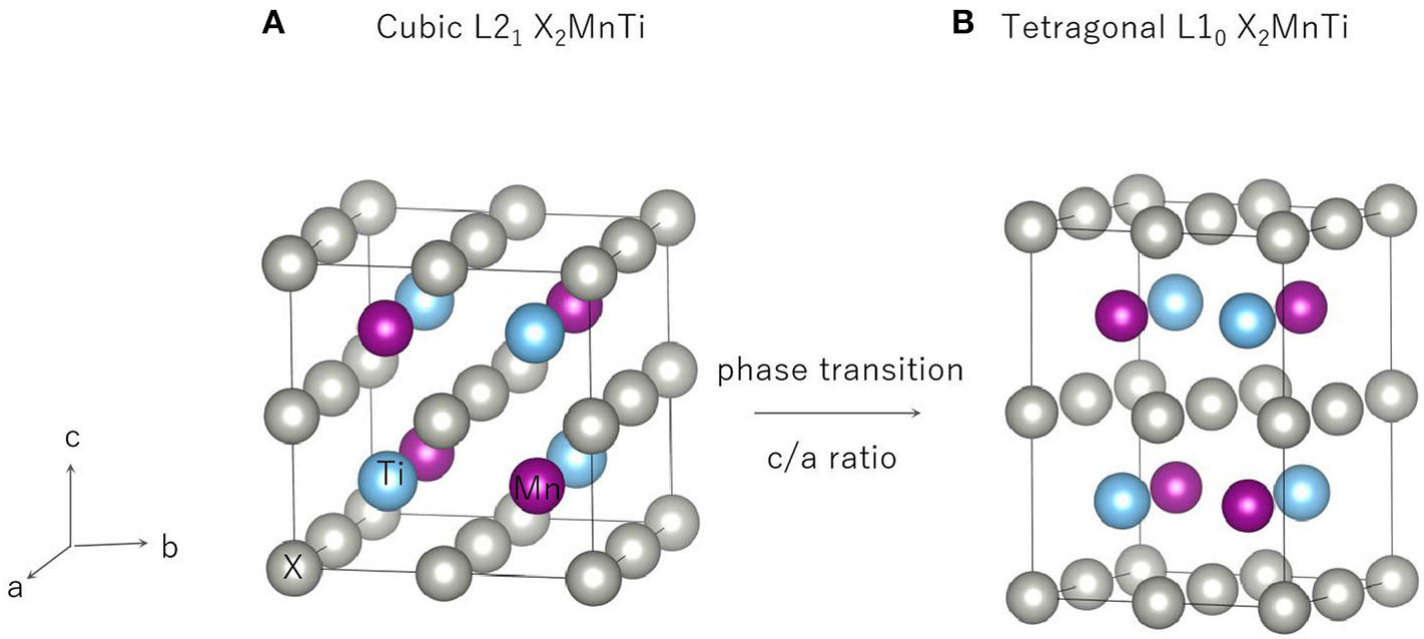
Cubic phase (L2_1_) **(A)** and tetragonal phase (L1_0_) **(B)** of X_2_MnTi compounds.

In this section, our research goal is to explore the possible competition between the L2_1_ (see Figure 4A) and L1_0_ (see Figure 4B) of the all-*d*-metal Heusler compounds X_2_MnTi. We used Bain paths to investigate the reversible transformation between the ordered L2_1_ and L1_0_ phases during tetragonal distortion. Bain paths (Alippi et al., 1997) have tetragonal states along the geometries which connect the bcc and fcc phases of a material. It is assumed that there is no cell volume change after applying deformation on the cubic phase, then the energy difference Δ*E* in respect to *c*/*a* ratios is calculated to estimate whether it is a stable or metastable phase. It is a commonly used method to predict reversible transformation, and a number of literature (Barman et al., 2007; Özdemir Kart et al., 2008; Qawasmeh and Bothina, 2012; Zeleny et al., 2014) have used this method to estimate shape memory effect in Heusler FSMAs, such as Ni_2_MnGa and Mn_2_NiGa.

**Figure 5:**
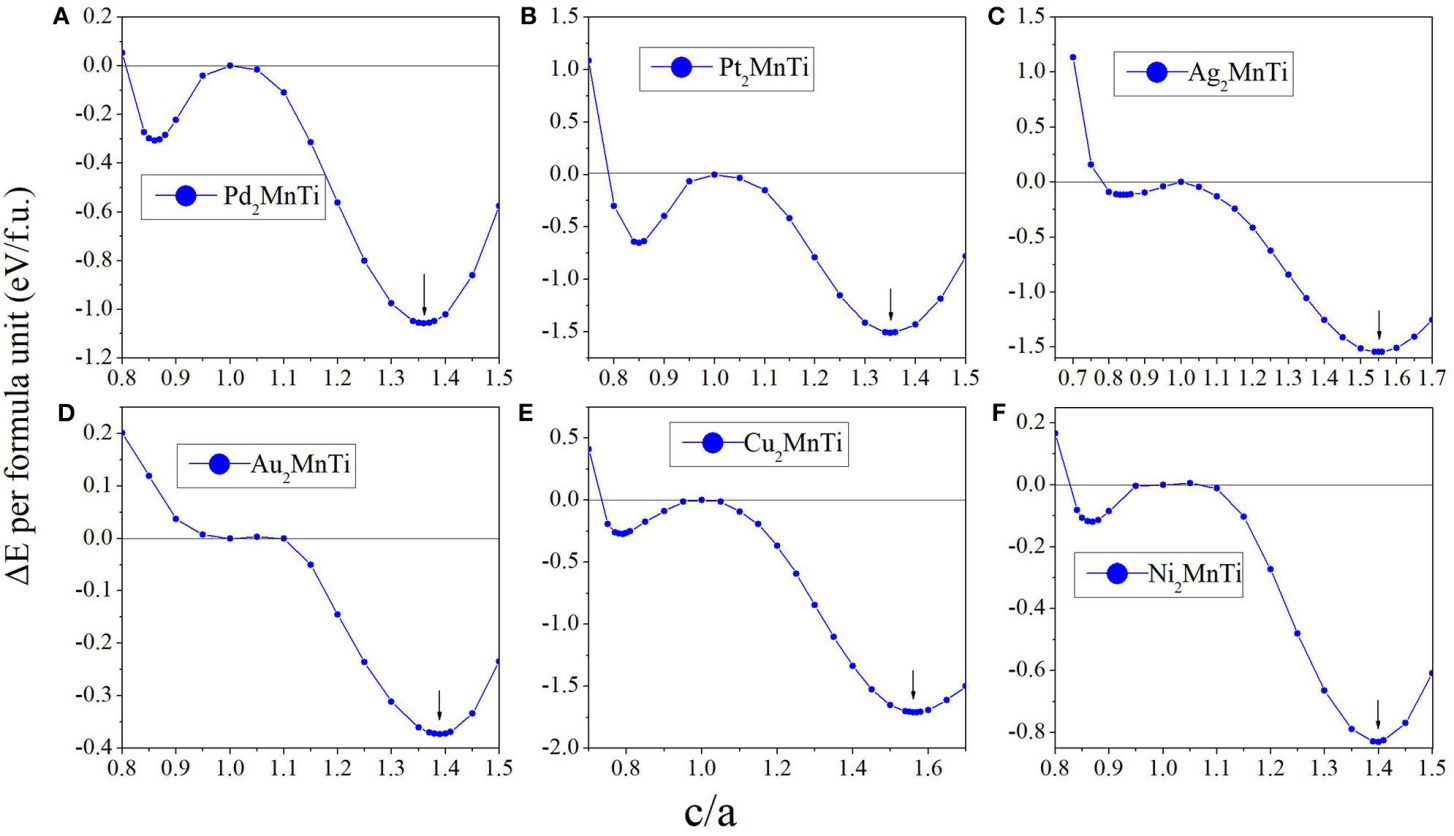
**(A–F)** Relationship between the *c*/*a* ratio and Δ*E* (Δ*E* = *E*_T_ – E_C_) for X_2_MnTi (X = Pd, Pt, Ag, Au, Cu, and Ni) compounds.

In Figure 5, we can see that all X_2_MnTi (X = Pd, Pt, Ag, Au, Cu, and Ni) compounds present a L2_1_-L1_0_ (possible martensitic transformation) under the effect of tetragonal distortion. In detail, we found that X_2_MnTi (except for X = Au) compounds have two local minimum energies, one locates at *c*/*a* < 1 and the other locates at *c*/*a* > 1. The value of the local minimum energy with *c*/*a* > 1 is lower than that of the local minimum energy with *c*/*a* < 1, which means that the local minimum energy with *c*/*a* > 1 is the most stable state. Moreover, when *c*/*a* < 1, the local minimum energy of X_2_MnTi (except for X = Au) is a metastable phase. For the Au_2_MnTi compound, there is only one local minimum energy, which locates at *c*/*a* >1. This local minimum energy is the most stable phase and there is no metastable structure during the cubic–tetragonal transformation. The *c*/*a* ratio values of the most stable tetragonal L1_0_ structure of these materials are summarized in **Table 2**.

**Figure 6:**
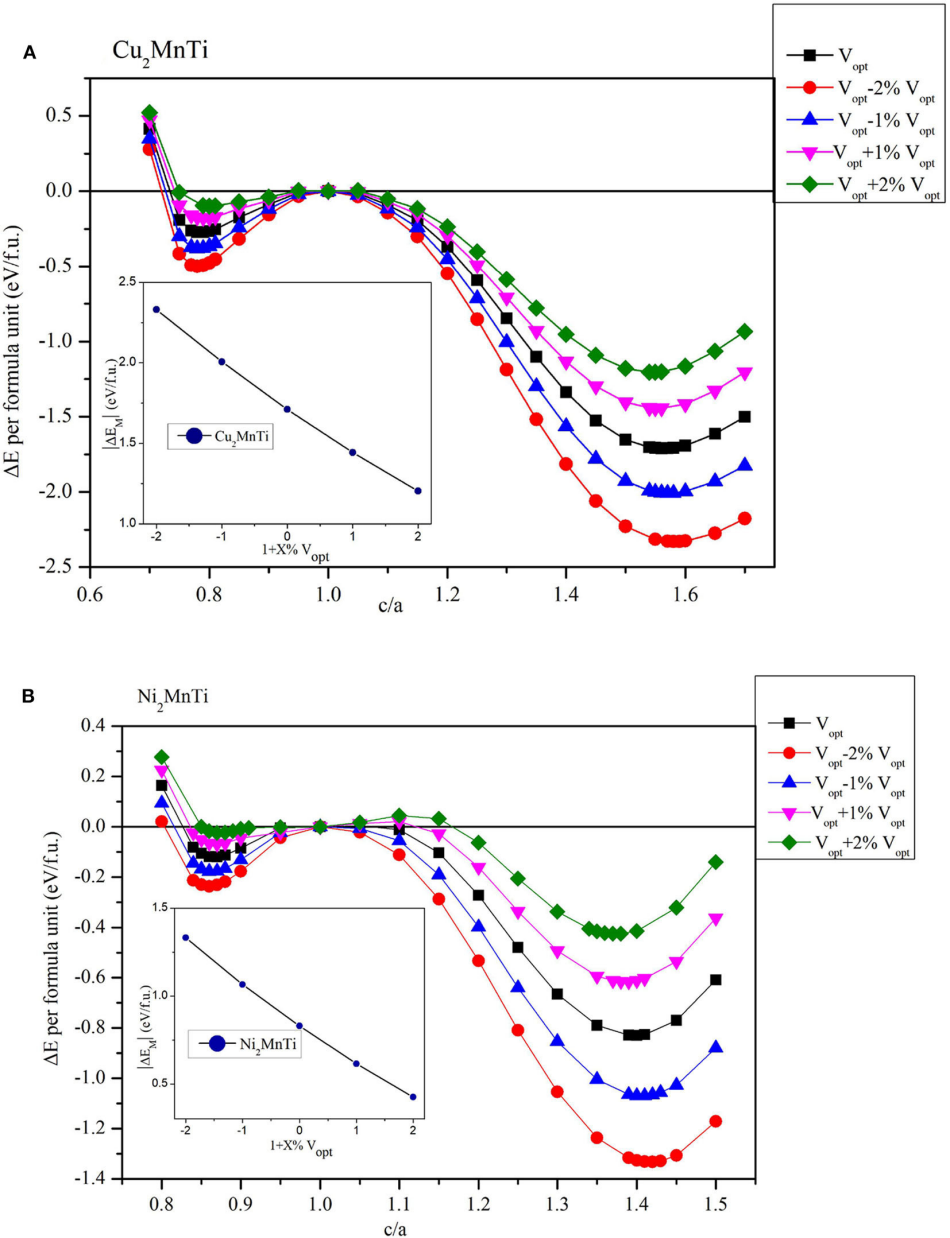
Relationship between *c*/*a* ratio and Δ*E* (Δ*E* = *E*_T_ – *E*_C_) with different volumes (insert figures: the relationship between the |Δ*E*_M_| and 1 + X%*V*_opt_) for **(A)** the Cu_2_MnTi compound and **(B)** the Ni_2_MnTi compound.

Next, we studied the influence of uniform strain on the competitiveness of the L2_1_ and L1_0_ structures. We give Cu_2_MnTi and Ni_2_MnTi as examples in Figure 6 for a detailed discussion.

By adjusting the lattice parameters of X_2_MnTi (X = Cu, Ni), their volume was changed between −2 and +2%, and then tetragonal distortion is applied to the cubic crystal structures. As shown in Figure 6, under the effect of tetragonal distortion, we can see that all substances with a volume of −2 and +2% still have two local energy minimums, which is the same as the optimized volume (*V*_opt_). However, the difference is that during the volume changes from +2 to −2%, the minimum value of local energy gradually becomes lower, that is, the L1_0_ state becomes more and more stable as the volume decreases.

In addition, it can be seen that the value of the *c*/*a* ratio for the most stable L1_0_ phase also changes with the volume changes. During the volume changes from −2% to +2%, the *c*/*a* ratio (for the most stable L1_0_ phase) gradually decreases. A smaller *c*/*a* ratio means a smaller degree of tetragonal distortion.

### The Calculated Density of States of All-*d*-Metal Heusler-Type Compounds X_2_MnTi

**Figure 7:**
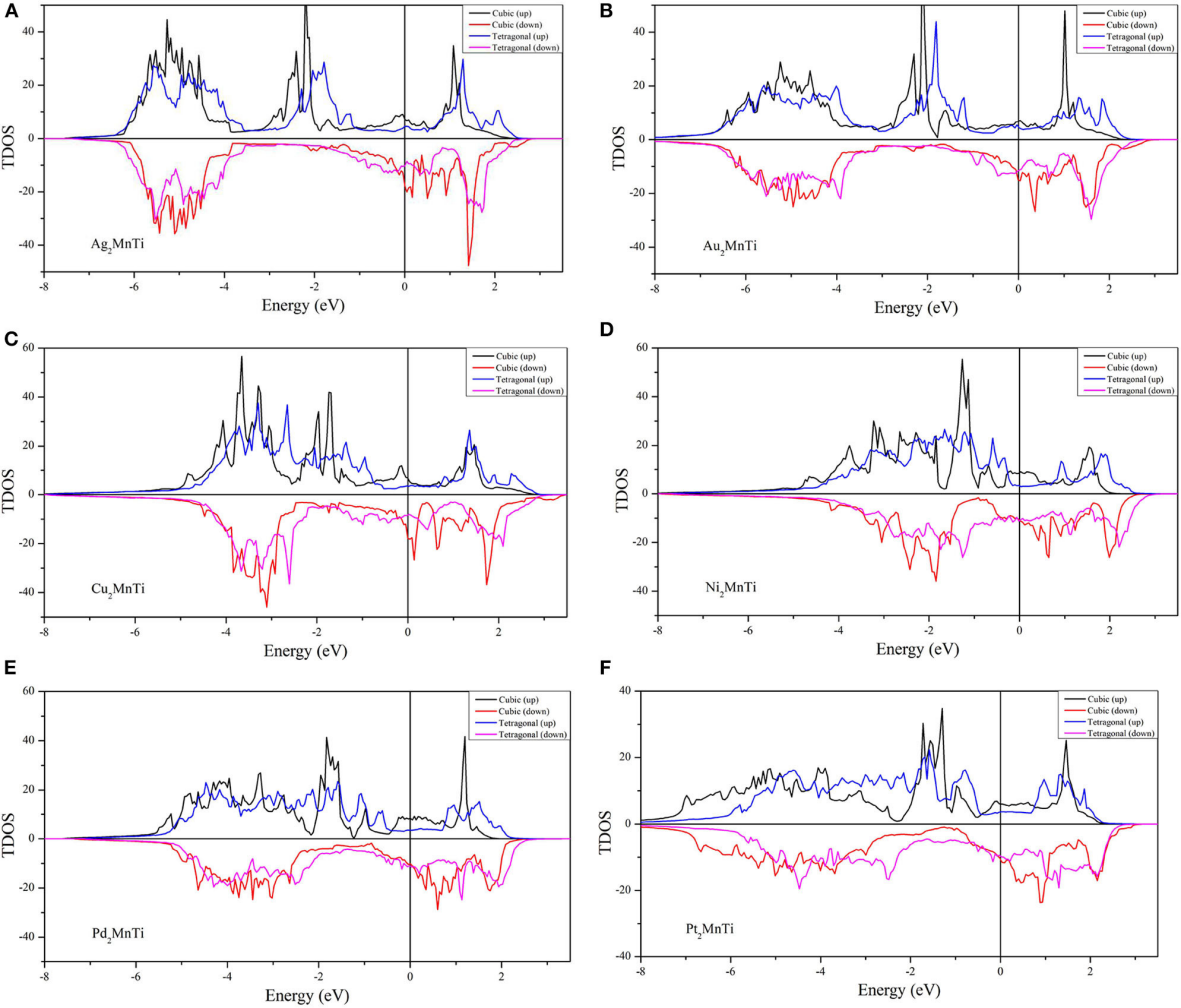
**(A–F)** Calculated TDOSs of X_2_MnTi (X = Pd, Pt, Ag, Au, Cu, and Ni) compounds in cubic and tetragonal phases.

**Figure 8:**
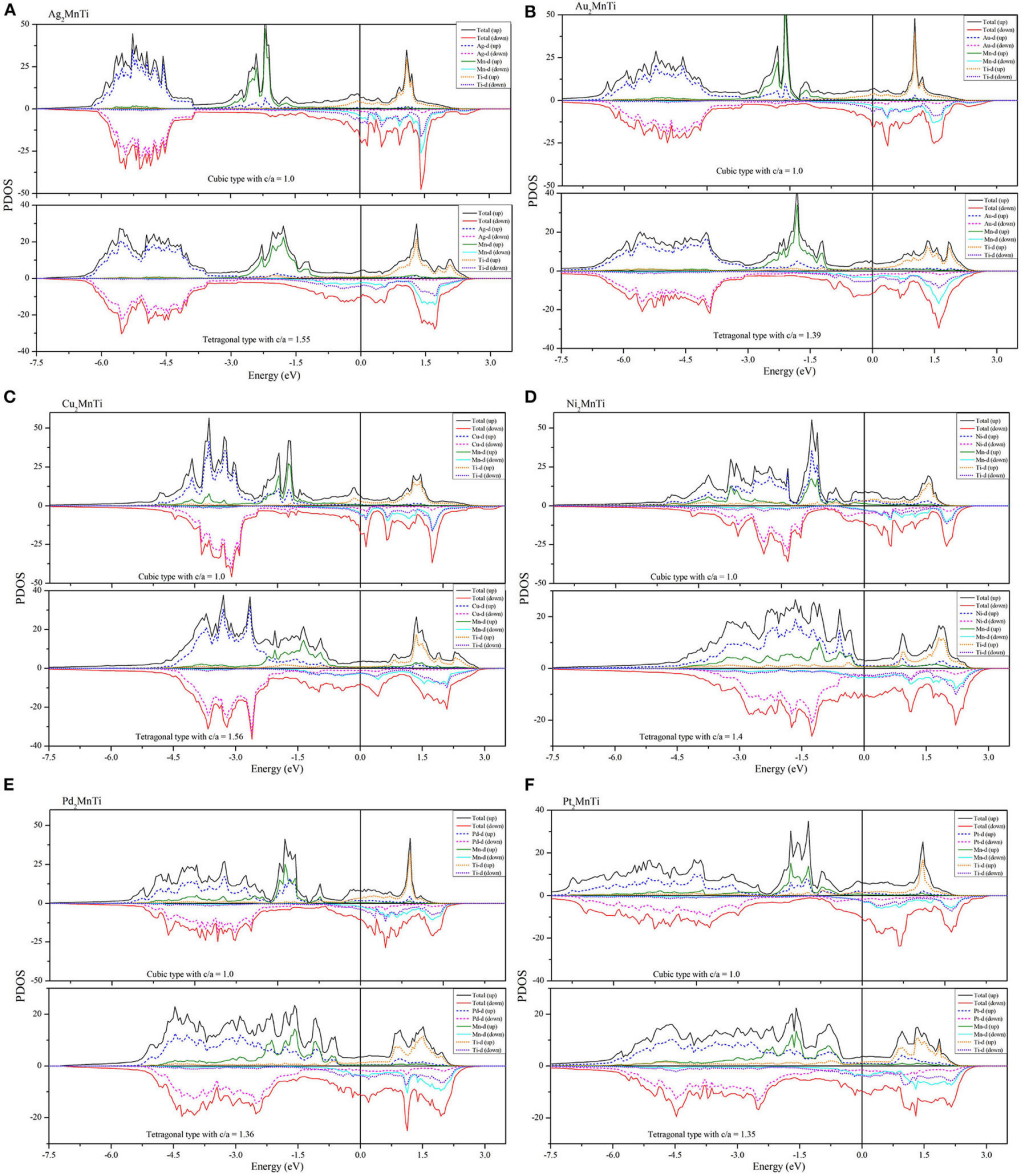
**(A–F)** PDOS diagrams of X_2_MnTi (X = Pd, Pt, Ag, Au, Cu, and Ni) compounds in cubic and tetragonal phases.

We calculated their total density of states (TDOSs) and partial state density of states (PDOSs) (Han et al., 2019b) in cubic and tetragonal states, respectively, and we plotted them in Figures 7, 8.

We found that the TDOS of tetragonal structure around the Fermi level is softer than the cubic TDOS. For example, we can find some strong peaks (see the red lines) near the Fermi level in the spin-down direction and some small peaks in the spin-up direction (see the black line) for the cubic phase (see Figure 7A). Note that one of the contributions to the total energy is the band energy $E_{\text{band}}=\int_{E_{\min}}^{E_{\text{F}}}\text{d}E\text{DOS}\left(E\right)E$; a reduction of the DOS near *E*_F_ in a tetragonal phase, in conjunction with the conservation of the integral for the number of valence electrons $N_{\text{V}}=\int_{E_{\min}}^{E_{\text{F}}}\text{d}E\text{DOS}\left(E\right)$, often leads to a lower band energy and, thus, to a lower total energy for the tetragonal phase than for the cubic phase. As shown in Figure 7, we can find that, under the effect of the tetragonal strain, the energy states near *E*_F_ tend to decrease or move to a high energy level. We give two more detailed explanations as follows: (1) as shown in Figures 7A,C–F, we can clearly observe that the local energy states (blue lines) near the Fermi level of the tetragonal L1_0_ phase are significantly lower than those of the cubic L2_1_ phase (black lines). Thanks to the TDOSs above, the total energy of the system will be released, resulting in cubic–tetragonal transformation; (2) as shown in Figures 7A,C, we find that the peaks (red line) of the cubic structure near the Fermi level disappeared in the tetragonal structure; however, a very small valley at the same energy (see the pink line) occurred. The peak to valley transition of TDOS near the Fermi level also proves that the tetragonal L1_0_ structure is more stable for X_2_MnTi compounds.

In Figure 8, we also show the PDOSs of each atom in the cubic and tetragonal phases. As shown in Figure 8A, the PDOS of the L2_1_-type Ag orbitals is almost located in the energy areas of −4.5 to −6 eV. In this region, the spin-up and spin-down PDOSs of Ag-d are almost symmetrical, reflecting that the contribution of Ag atoms to the total magnetism is relatively small. In the energy range from −1.5 to −4 eV, two large energy peaks, which come from the Mn-d orbitals, can be found in the spin-up channel; however, the DOS in the spin-down is nearly flat. Above the Fermi level, the TDOS in the spin-up channel is coming from the Ti-d orbital, and the TDOS in the spin-down channel is arising from the hybridization between the Ti-d and Mn-d orbitals. A similar phenomenon can also be found in L2_1_-type Au_2_MnTi as shown in Figure 8B.

As shown in Figures 8C–F, some large peaks can be obviously found below the Fermi level in the spin-up channel for the L2_1_-type X_2_MnTi (X = Ni, Cu, Pd, Pt) alloys. The formation of these peaks owes to the hybridization between X-d and Mn-d orbitals. For Ni_2_MnTi, the energy peak of the Ni-d orbital is larger than that of the Mn-d orbital around −1.2 eV; however, for the other three cases, the energy peaks of the X-d orbital are smaller than those of the X-d orbitals. Above the Fermi level, the TDOS is mainly coming from the Mn-d orbital in the spin-up channel and from the Mn-d and X-d orbitals in the spin-down channel.

As shown in Figure 8, near the Fermi level, PDOSs of the Mn atom will produce a strong spin splitting in two spin channels, and then result in strong magnetism. Thus, the total magnetism of X_2_MnTi is mainly coming from the Mn atoms. We also exhibit the total and atomic magnetic moments in Tables 1, 2.

**Table 1:** Total and atomic magnetic moments for cubic L2_1_ type X_2_MnTi (X = Pd, Pt, Cu, Ni, Ag, Au).

**Compounds X_**2**_MnTi**	**Mt (μ_B_/f.u.)**	**M_**Mn**_ (μ_B_)**	**M_**Ti**_ (μ_B_)**	**M_**X-1**_ (μ_B_)**	**M_**X-2**_ (μ_B_)**
Pd_2_MnTi	3.9295	3.792	−0.108	0.123	0.123
Pt_2_MnTi	4.17525	3.774	0.008	0.157	0.157
Cu_2_MnTi	3.4385	3.415	−0.117	0.069	0.069
Ni_2_MnTi	3.90075	3.372	−0.098	0.313	0.313
Ag_2_MnTi	3.45975	3.79	−0.376	0.022	0.022
Au_2_MnTi	3.60475	3.76	−0.215	0.03	0.03

**Table 2:** Total and atomic magnetic moments, and c / a ratio for tetragonal L1_0_ type X_2_MnTi (X = Pd, Pt, Cu, Ni, Ag, Au).

**Compounds X_**2**_MnTi**	**Mt (μ_B_/f.u.)**	**M_**Mn**_ (μ_B_)**	**M_**Ti**_ (μ_B_)**	**M_**X-1**_ (μ_B_)**	**M_**X-2**_ (μ_B_)**	**c/a**
Pd_2_MnTi	3.18775	3.446	−0.526	0.134	0.134	1.36
Pt_2_MnTi	3.3185	3.429	−0.396	0.143	0.143	1.35
Cu_2_MnTi	1.77925	2.487	−0.722	0.007	0.007	1.56
Ni_2_MnTi	3.092	2.799	−0.415	0.323	0.323	1.4
Ag_2_MnTi	2.01	3.198	−1.147	−0.02	−0.02	1.55
Au_2_MnTi	2.42525	3.352	−0.919	−0.004	−0.004	1.39

**Figure 9:**
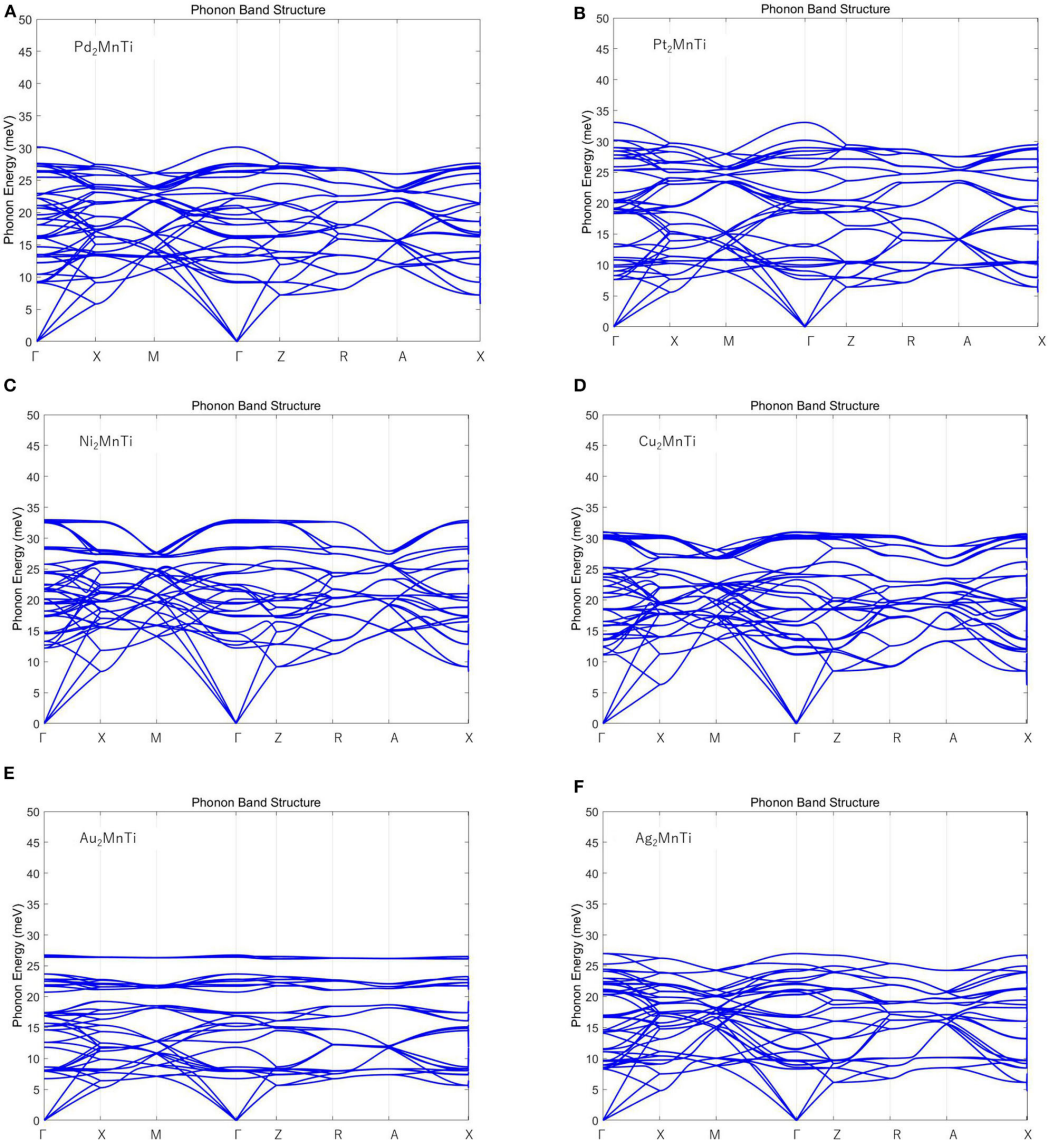
**(A–F)** Calculated phonon energy of X_2_MnTi (X = Pd, Pt, Ag, Au, Cu, and Ni) with tetragonal L1_0_ phase.

Finally, we calculated the phonon spectrum of the X_2_MnTi (X = Pd, Pt, Ag, Au, Cu, and Ni) tetragonal L1_0_ phase by means of the force-constants method using Nanodcal code and the results are collected in Figure 9. In Figure 9, we can clearly see that there are no virtual frequencies in the phonon spectrum of the X_2_MnTi, and the absence of virtual frequencies further confirms that their tetragonal L1_0_ states are theoretically stable. Unfortunately, the possible L2_1_-L1_0_ phase transition of X_2_MnTi (X = Pd, Pt, Ag, Au, Cu, and Ni) has not been studied experimentally, and therefore, a comparison between the theoretical and experimental results cannot be shown in the current work. However, this investigation can help in understanding the physics in all-*d*-metal alloys. Moreover, we would like to point out that Bain paths are a sophisticated way to investigate the reversible transformation between the L2_1_ and L1_0_ phases during the tetragonal distortion. This method has been widely used to design new MSMAs, and some designed MSMAs have been experimentally verified, such as the Mn–Ni–Co–Ti system.

## Conclusions

In this study, we investigated the phase transition and electronic structures of X_2_MnTi (X = Pd, Pt, Ag, Au, Cu, and Ni) compounds based on first-principle calculations. First, we examined the L2_1_ and XA competition of Heusler compounds X_2_MnTi (X = Pd, Pt, Ag, Au, Cu, and Ni). The results show that the L2_1_ type is the most stable ordered structure for these newly designed materials. Based on the L2_1_ structure, we also compared the total energies of the L2_1_ X_2_MnTi system with different magnetic states, i.e., FM and AFM. We found that these compounds have lower ground state energy when in the FM state, that is, the most stable state of X_2_MnTi is the FM state under the L2_1_ structure. Subsequently, we studied the possible tetragonal transformation of Heusler compounds X_2_MnTi (X = Pd, Pt, Ag, Au, Cu, and Ni) and found that these materials all feature a stable L1_0_ tetragonal phase. The energy difference Δ*E*_M_ (*E*_T_ – *E*_C_) in X_2_MnTi can be adjusted by a uniform strain. By analyzing the DOS diagram, it can be found that the magnetic moment of X_2_MnTi mainly comes from Mn atoms, which is due to their strong spin splitting around *E*_F_. The lower TDOS of the tetragonal L1_0_ state near the Fermi level can be used to explain the stability of the tetragonal L1_0_ state of X_2_MnTi. Finally, we gave the phonon spectra of the tetragonal L1_0_ phase of X_2_MnTi (X = Pd, Pt, Ag, Au, Cu, and Ni) to further prove their stability.

## Data Availability Statement

The raw data supporting the conclusions of this article will be made available by the authors, without undue reservation.

## Author Contributions

MW: conceptualization, methodology, software, and writing—original draft preparation. FZ and RK: software and writing—original draft preparation. MK and XW: supervision. All authors contributed to the article and approved the submitted version.

## Conflict of Interest

The authors declare that the research was conducted in the absence of any commercial or financial relationships that could be construed as a potential conflict of interest.
